# Single-Cell RNA-Seq Recognized Key Genes for Metastasis and Macrophage Infiltration in Colorectal Cancer

**DOI:** 10.1155/humu/9488531

**Published:** 2025-05-15

**Authors:** Juan Shi, Peiming Zheng, Libo Ouyang, Facai Cui

**Affiliations:** Department of Clinical Laboratory, Henan Provincial People's Hospital, Zhengzhou, China

**Keywords:** colorectal cancer, macrophages, metastasis, single-cell sequencing, ZFAND2A

## Abstract

Colorectal cancer (CRC) is one of the most common malignancies in the world. However, the main causes of metastasis and immune cell infiltration in CRC are still unclear. This experiment was conducted to identify the key genes of metastasis and macrophage infiltration in CRC according to single-cell sequencing (scRNA-seq) data. By analyzing the data of GSE261012 and GSE234804 in the Gene Expression Omnibus (GEO) database, the key node genes for the stages of tumorigenesis, epithelial–mesenchymal transition, and metastasis of CRC were found. These genes were modeled by lasso regression by The Cancer Genome Atlas (TCGA) database, and ZFAND2A was identified as a key gene for metastasis and macrophage infiltration in CRC. Finally, the specific function of ZFAND2A in cancer cell activity was explored in vitro by qRT-PCR, WB analysis, CCK-8, and transwell assay. The specific function of ZFAND2A in macrophage polarization was explored in vitro by qRT-PCR, ELISA, and flow cytometry. We identified crucial gene expression in the entire process of CRC tumor progression, including tumorigenesis, epithelial–mesenchymal transition, and metastasis. Ten thousand six hundred and thirty-seven genes were determined as genes associated with tumor progression and metastasis. Among them, six genes were identified to be related to CRC prognosis. The results of TCGA data indicated that ZFAND2A showed lower expression in tumors and was related to a good prognosis of CRC. Overexpression of ZFAND2A inhibits the proliferation and migration of CRC cells. Additionally, there was a correlation between ZFAND2A expression and macrophage infiltration. Increasing ZFAND2A promotes M1 polarization in macrophages. Our findings provide new potential biomarkers for the metastatic mechanisms and prognosis of CRC. In addition, ZFAND2A is expected to become a potential therapeutic target for CRC.

## 1. Introduction

Colorectal cancer (CRC) is the third leading cause of cancer-related morbidity and mortality worldwide [1]. Distant metastasis is the leading cause of CRC-related deaths, with the liver and lungs being the most commonly affected organs [2]. Surprisingly, up to 90% of CRC-related deaths are due to distant invasion and metastasis [3]. Thus, distal metastasis of CRC is the greatest challenge encountered in its clinical management. To overcome the challenge of CRC distal metastasis, it is crucial to clarify the mechanisms underlying CRC aggravation and metastasis.

Metastatic processes are multistep events [4, 5]; many factors regulate metastasis, including cell-intrinsic factors (genetic abnormalities, tumor cell heterogeneity, and epithelial–mesenchymal transition [EMT]) and the tumor microenvironment (TME) [6, 7]. Among these, cell-intrinsic factors are important factors that cause metastasis. Recent studies have shown that metastasis arise from subpopulations of cancer stem or progenitor cells. Distinguishing these metastasis-initiating cells is important to prevent CRC metastasis. For example, a unique population of epithelial tumor cells was identified and expressed genes associated with poor prognosis in patients with CRC [8]. Tumor-associated macrophages (TAMs) are a major component of the TME and are usually associated with tumor metastasis in human cancers [9]. Chen et al. found that CRC cells regulate the EMT program by promoting macrophage recruitment, which enhances CRC cell migration and invasion [10]. SPON2 drives M2 macrophage infiltration to promote CRC tumor growth and metastasis [11]. The above studies illustrate that exploring the initiating cells and TME of CRC metastasis is key to addressing CRC malignant metastasis. As an innovative technology, single-cell sequencing (scRNA-seq) was used for discerning cellular and molecular heterogeneities of CRC [12–14]. Moreover, scRNA-seq could also define a continuum of cellular states and compositional changes that lead to the transformation of malignant polyps into CRC [15]. ScRNA-seq also described the immune environment of primary CRC [16, 17], but studies on the dynamic processes of CRC initiation, cellular heterogeneity, EMT, and CRC metastasis induced by the TME are rarely reported.

**Figure 1:**
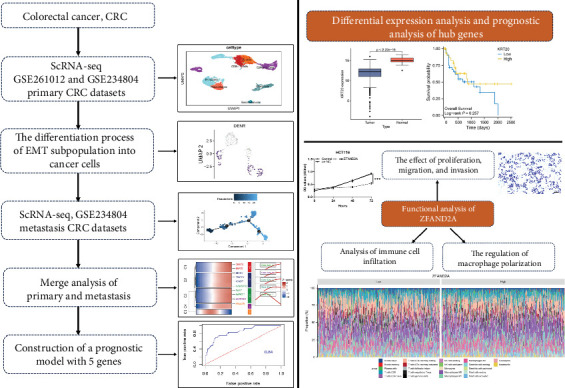
The overall flow diagram of this study.

In this study, key genes for heterogeneous changes in CRC were explored according to scRNA-seq datasets. These genes included key node genes involved in epithelial tumor cell evolution, differentiation of epithelial–mesenchymal subpopulations to tumor cells, and tumor metastasis. Finally, these nodal genes were integrated to determine the gene modules associated with CRC progression and metastasis. Among them, ZFAND2A was identified as a key signature gene during CRC progression via The Cancer Genome Atlas (TCGA) database. Further analyses revealed a correlation between ZFAND2A expression and macrophage infiltration. ZFAND2A is a critical therapeutic target for CRC metastasis. The overall methodology used in this research is illustrated in Figure 1.

## 2. Materials and Methods

### 2.1. Data

The GSE261012 and GSE234804 datasets were obtained from the Gene Expression Omnibus (GEO) (https://www.ncbi.nlm.nih/). The GSE234804 dataset contained scRNA-seq data from three patients with primary CRC and six patients with liver metastasis, and GSE261012 contains scRNA-seq data from one CRC patient. The test platform used was a GPL24676 Illumina NovaSeq 6000 (*Homo sapiens*).

### 2.2. ScRNA-Seq Data Processing

CRC scRNA-seq data was read from GSE217774 and GSE234804 by the R package “Seurat,” and Seurat objects were created [18]. The cells were isolated using the following criteria [19, 20]: cells (containing less than 50 genes) and cells (expressing < 3 genes) were excluded. Cells (containing > 5% of mitochondrial genes) were removed. FindVariableFeatures and NormalizeData functions were used to identify the top 2000 highly variable genes, followed by principal component analysis (PCA) on all samples. Further analysis was performed on the top 50 principal components. The clustered cells on a two-dimensional map was displayed by the uniform manifold approximation and projection (UMAP) dimensionality reduction technique. Next, differentially expressed genes (DEGs) between clusters were identified by the Wilcoxon–Mann–Whitney test and the “FindAllMarkers” function in the “Seurat” package. Genes with |log_2_ (fold change) |> 2 and adjusted *p* < 0.01 were identified as cellular markers.

### 2.3. Cellular Annotation

Automatic annotation [21]: The types of population-differentiated genes in the Seurat object were analyzed by the “Hspe. The human annotation” file in the “SingleR” package [22]. Manual annotation [23]: Tumor and epithelial cells were divided through the CopyKAT package, and Seurat subsets were extracted for tumor cell splitting by subset function. Meanwhile, the doubled and aneuploid conditions of all cell subpopulations in the Seurat file were identified using inferred copy number variation (InferCNV) software, which was used to observe the copy number variation of multiple cell types in the process of CRC development and aggravation.

### 2.4. Functional Enrichment Analysis

Firstly, the gene ID in different populations were converted by “clusterProfiler” function package in R [24]. Enrichment of Kyoto Encyclopedia of Genes and Genomes (KEGG) and Gene Ontology (GO) was performed by the “enrich” function, and bubble plots of biosignals were enriched by the “barplot” function. In addition, enrichment analysis of key genes involved in tumor aggradation dynamic process was performed using the Metascape platform (http://metascape.org) [25]. The number of min overlaps and min enrichment was 3, and the cutoff value was *p* < 0.05.

### 2.5. Pseudotime Trajectories

CDS objects were constructed and normalized by data (Seurat expression matrix), cell metadata, gene annotation in the Monocle 3 package, and “preprocess-CDS” function. After generating a UMAP with the “reduce-dimension” function [26], learning the developmental trajectory using the “cluster-cells” and “learn-graph” functions, “primary tumor cells and benign bone tissue” were set as root cells. Next, the genes associated with the developmental trajectory were examined and visualized by “plot_cells” function.

### 2.6. Construction and Validation of a Prognostic Model

RNA-Seq and clinical data were obtained from the TCGA-COAD dataset in the TCGA database. The TCGA-related data were processed using the “dplyr” package [27] and visualized using the “ggplot2” package [28]. The training set included 152 patients with 10,637 gene expressions from TCGA database (https://portal.gdc.cancer.gov/). The lasso modeling and the achievement of area under the curve (AUC) values were processed using the “glmnet” and “ROCR” packages [29]. Credible gene was screened through the “choose_gene_min” and “choose_gene_1se” functions. Survival curves was constructed by R package “survminer” [30].

### 2.7. Random Survival Forest (RSF) Analysis

The significance of the associated genes was assessed by R software “randomForestSRC” with RSF algorithm [31]. Genes (relative relevance > 0.3) were screened for the final signature.

### 2.8. Immunocyte Infiltration Analysis

The proportions of 22 different infiltrating immune cell types were determined by R package “CIBERSORT” on RNA-Seq data [32]. And the correlation between risk scores and tumor-infiltrating immune cells was analyzed by Pearson's correlation coefficients.

### 2.9. Cell Culture

Human macrophage THP-1 (TIB-202) and CRC cell lines (HT29 [HTB-38] and HCT116 [CCL-247]) were purchased from ATCC (Manassas, Virginia, United States). THP-1 and HT29 cells were cultured in RPMI 1640 medium (Gibco, Grand Island, New York, United States) containing 10% fetal bovine serum (FBS; Gibco), 100 U/mL penicillin, and 100 *μ*g/mL streptomycin (Invitrogen, Waltham, Massachusetts, United States) at 37°C and 5% CO_2_. HCT116 cells were cultured in Dulbecco's modified Eagle's medium (DMEM; Gibco, Grand Island, New York, United States), supplemented with FBS and penicillin/streptomycin at 37°C and 5% CO_2_. Phorbol-12-myristate-13-acetate (10 ng/mL, PMA, Sigma, St. Louis, Missouri, United States) was used to induce THP-1 differentiation for 24 h.

### 2.10. Cell Transfection

PcDNA3.0 (GenePharma, Shanghai, China) was used to construct overexpression ZFAND2A (oe-ZFAND2A) and empty vector plasmids (oe-NC). Full-length human ZFAND2A cDNA sequences were obtained from the National Center for Biotechnology Information (NCBI) reference sequence (NM_001365383.1). Transfection of the plasmid into CRC cells was achieved by Lipofectamine 3000 reagent (Invitrogen, Waltham, Massachusetts, United States).

### 2.11. Cell Counting Kit-8 (CCK-8)

CRC cells were cultured with 100 *μ*L medium in 96-well plates for 0, 24, 48, and 72 h. Following the treatment, 10 *μ*L of the CCK-8 reagent (Beyotime Biotechnology, Shanghai, China) was added. After 4 h at room temperature, the spectrophotometer (Multiscan MK3, Thermo Fisher Scientific, Waltham, Massachusetts, United States) was conducted to measure the absorbance of the samples at 450 nm at different time points.

### 2.12. Transwell Assay

For migration assay, CRC cells were resuspended in 200 *μ*L of FBS-free medium (1 × 10^5^ cells) and added to the top chamber (BD Bioscience, Franklin Lakes, New Jersey, United States) of transwell (8 *μ*m wells). The lower chamber was added with the medium supplemented with 10% FBS. After 24 h, cells were fixed, stained, and counted under a microscope. For invasion assay, similar to the migration assay, an upper chamber was coated with matrix gel (BD Bioscience, Franklin Lakes, New Jersey, United States).

### 2.13. Quantitative Reverse Transcription PCR (qRT-PCR)

RNA was extracted with the TRIzol reagent (Takara, Tokyo, Japan); then, 4 *μ*g of total RNA was converted to cDNA by MultiScribe Reverse Transcriptase (Applied Biosystems, Foster City, California, United States), followed by amplifying with Fast SYBR Green Master Mix (Applied Biosystems, Foster City, California, United States). The amount of RNA extracted using StepOnePlus was measured using qRT-PCR. The mRNA expression was quantified using the 2^-*ΔΔ*Ct^ method. Primer sequences are detailed in Table S1.

### 2.14. Western Blotting (WB) Analysis

The whole protein was extracted by RIPA lysis buffer (Beyotime Biotechnology), and the protein concentration was determined by a bicinchoninic acid (BCA) protein assay kit (HyClone-Pierce, Guangzhou, China). Polyvinylidene difluoride (PVDF) membranes were used to transfer sodium dodecyl sulfate (SDS)–10% polyacrylamide gels that had been loaded with 20 *μ*g protein extract. Membranes were immersed in 5% (w/v) skim milk for 2 h. After that, each membrane was probed with primary antibodies, anti-ZFAND2A (HPA019469, 0.1 *μ*g/mL, Sigma, St. Louis, Missouri, United States) and anti-GAPDH (10494-1-AP, 1:5000, Proteintech, Wuhan, China), and kept at 4°C for an extra night of incubation, followed by horseradish peroxidase (HRP)–labeled secondary antibodies (SA00001-15, 1:2000, Proteintech, Wuhan, China) for 1 h at room temperature. The membrane was then cleaned and investigated using the enhanced chemiluminescence (ECL, Millipore, Billerica, Massachusetts, United States) detection method. Software for ImageJ analysis was used for estimation.

### 2.15. Enzyme-Linked Immunosorbent Assay (ELISA)

IL-10 and TNF-*α* expression were analyzed in the macrophage supernatant to determine macrophage polarization status by IL-10 ELISA kit (R&D Systems, Minneapolis, Minnesota, United States) and TNF-*α* ELISA kit (Abcam, Cambridge, United Kingdom) according to the manufacturer's instructions.

### 2.16. Flow Cytometry

Cells were stained with anti-CD68-FITC, anti-CD80-APC, and anti-CD86-PE (BD Biosciences, San Jose, California, United States), followed by staining fluorophore-conjugated secondary antibodies for flow cytometry analysis.

### 2.17. Statistical Analysis

The experimental data was represented as mean ± standard deviation (SD). SPSS software (Version 26.0; SPSS Inc., Chicago, Illinois, United States) was conducted for statistical analysis. Unpaired Student's *t*-test was conducted to compare two groups. One-way ANOVA was used in statistical studies involving multiple group comparisons, and Tukey's post hoc test was then applied. Statistical significance was established (*p* < 0.05).

## 3. Results

### 3.1. ScRNA-Seq Revealed Nodal Gene Modules for Epithelial Cell Evolution During CRC Carcinogenesis

**Figure 2:**
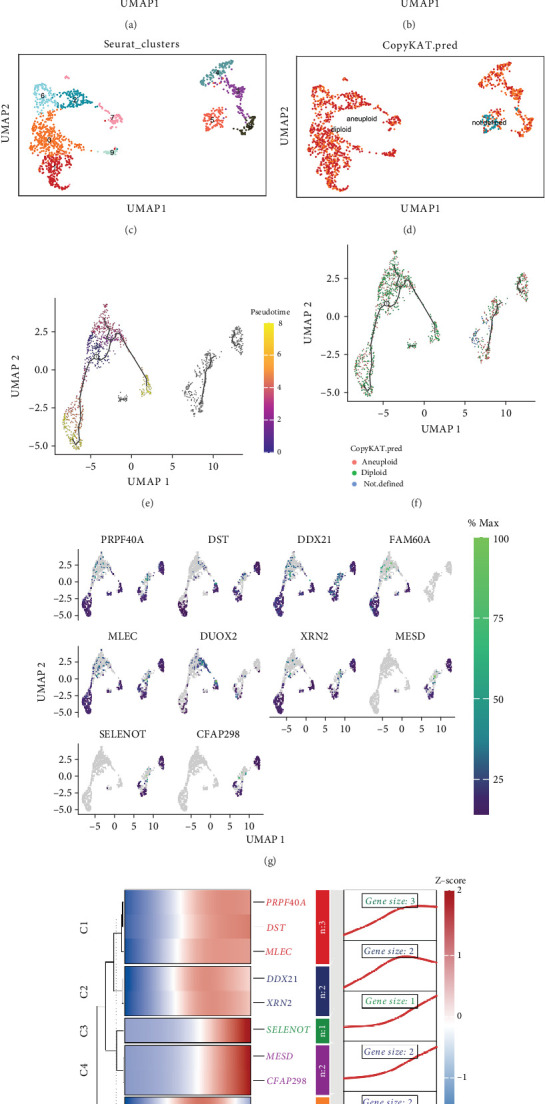
ScRNA-seq revealed nodal gene modules for epithelial cell evolution during CRC carcinogenesis. (a) UMAP visualization of cells from four primary CRC patients. Cells were categorized into seven cell types including epithelial cells, B cells, macrophages, monocytes, CD8^+^ T cells, endothelial cells, and oblasts. (b) UMAP visualization after extraction of epithelial cell subpopulations. (c) UMAP visualization of epithelial cell subpopulations was categorized into 10 clusters. (d) UMAP visualization of epithelial cell subpopulations was categorized into aneuploid and diploid subpopulations based on CopyKAT. (e, f) Trajectory analysis of aneuploid and diploid subpopulations using Monocle 3. (g) The expression of top 10 genes in aneuploid and diploid subpopulations. (h) Heatmap of top 10 genes for pseudotime trajectory analysis.

We downloaded scRNA-seq data from four primary CRC from the GEO databases GSE261012 and GSE234804. First, after parameter settings, 7944 cells were used for further analysis (Figure S1(a) and S1(b)). Second, the top 2000 highly variable genes were identified, and then, 10 DEGs in isolated cells would be the focus of further research (Figure S1(c)). According to PCA, 20 single-cell samples were scattered and showed a logically distributed pattern (Figure S1(d)) and varying scores across the multiple dimensions (Figure S1(e)). Seven cell clusters were obtained, including epithelial cells, B cells, macrophages, monocytes, CD8^+^ T cells, endothelial cells, and fibroblasts (Figure 2a). Further extraction of the epithelial cell subpopulations was followed by clustering and division. The results revealed 10 cell subpopulations (Figure 2b,c). Single-cell transcriptome data of epithelial cells were analyzed using CopyKAT, and aneuploid and diploid subpopulations of epithelial cells were successfully identified (Figure S1(f) and Figure 2d). The diploid subpopulation could be considered normal epithelial cells, while the aneuploid subpopulation was considered tumor cells. We conducted a single-cell pseudotime trajectory analysis of all epithelial cells. The results showed that diploid normal epithelial cells gradually evolved into aneuploid tumor cells over time (Figure 2e,f). In addition, we identified crucial genes during cellular evolution from epithelial cells to tumor cells, as shown in the UMAP plot (Figure 2g). Subsequently, these variant genes were subjected to pseudoannotation and developmental trajectory heatmap analysis (Figure 2h). The results of the above analysis revealed nodal gene modules of epithelial cell evolution during CRC.

### 3.2. ScRNA-Seq Revealed Nodal Genes in the Differentiation of Epithelial–Mesenchymal Subpopulations to CRC Tumor Cells

**Figure 3:**
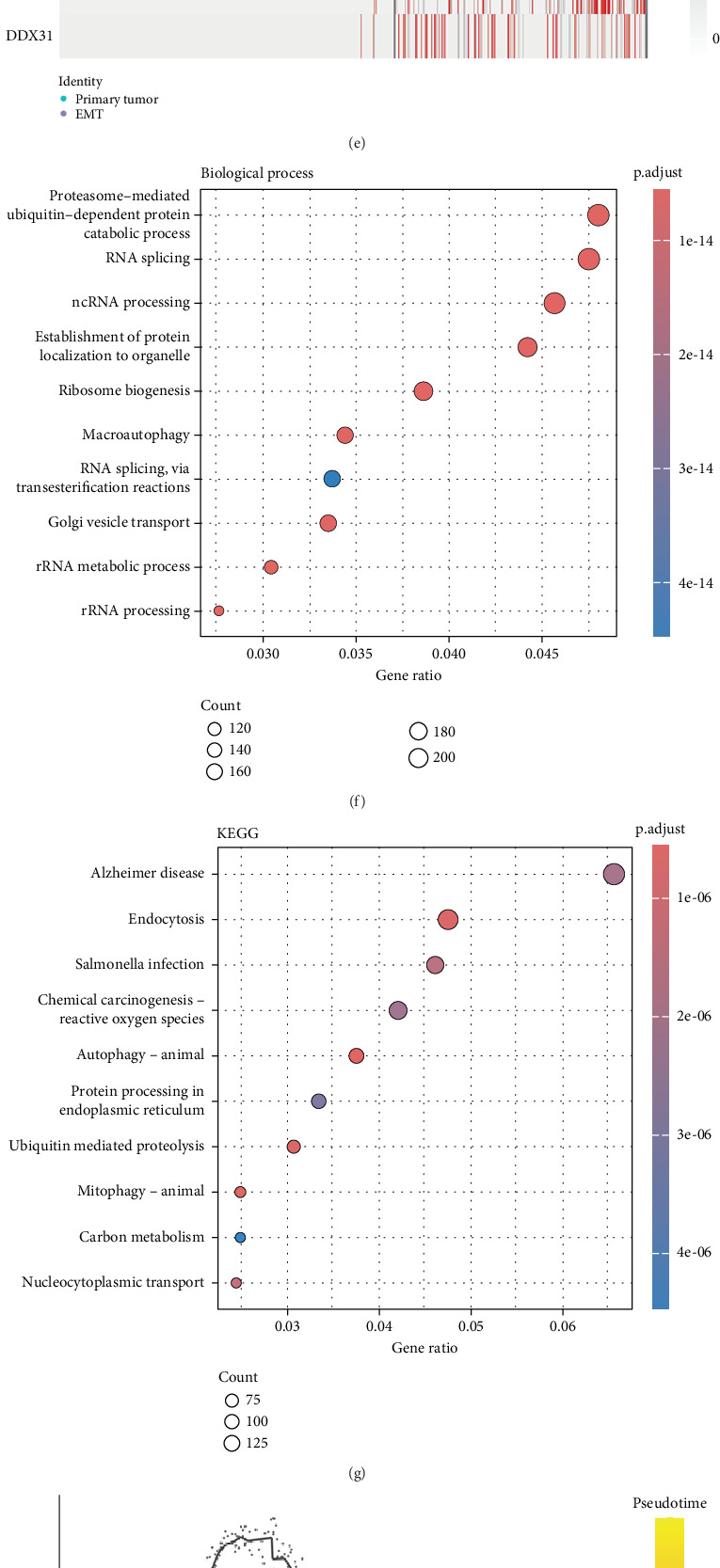
ScRNA-seq revealed nodal genes in the differentiation of epithelial–mesenchymal subpopulations to CRC tumor cells. (a) UMAP visualization of the remaining aneuploid cells. Cells were categorized into categorized into aneuploid and diploid subpopulations based on CopyKAT (right). (b) Heatmap of top 7 EMT gene expression level in different cell clusters. (c) A violin plot of EMT gene expression level in primary tumor clusters and EMT clusters. (d) UMAP visualization of primary tumor and EMT cells. (e) Heatmap of top 10 DEGs between primary tumor and EMT cells. (f, g) KEGG and GO enrichment analyses of DEGs between primary tumor and EMT cells. (h) Trajectory analysis of aneuploid and diploid subpopulations using Monocle 3. (i) Analysis of the developmental trajectory of top 10 genes associated with tumor epithelial–mesenchymal evolution. (j) Heatmap of top 10 genes for pseudotime trajectory analysis.

During the analysis of the epithelial-to-tumor evolution, we first subset diploid cells (Figure 3a). According to a previous report, the partial epithelial-to-mesenchymal transition (p-EMT) gene emerged as a biomarker for EMT [33]. We examined the expression of EMT marker genes in aneuploid cell populations and observed that the expression levels were higher in 2, 6, 7, and 8 cell populations (Figure 3b,c). Therefore, we defined 2, 6, 7, and 8 clusters as EMT clusters and other aneuploid cells as tumor clusters that were exhibited in the UMAP plot (Figure 3d). Furthermore, we compared the gene expression in EMT and aneuploid cells. A total of 5576 DEGs associated with EMT were identified and exhibited the top 10 DEGs in the heatmap (Figure 3e). To analyze the biological enrichment of DEGs, we enriched DEGs in the GO database, which refers to RNA splicing related to cancer cell death, ribosome biogenesis inherited mutations, and elevated cancer risk. KEGG was mainly enriched for endocytosis signaling, which reported effects in the immune environment of tumors (Figure 3f,g). We established the developmental trajectory of the tumors and EMT cells (Figure 3h). Crucial genes during cellular evolution from EMT to tumor cells are shown in the UMAP plot (Figure 3i). A heatmap exhibited the top 10 crucial genes involved in cellular evolution (Figure 3j).

### 3.3. ScRNA-Seq Uncovered Key Genes for Metastasis in CRC Tumors

**Figure 4:**
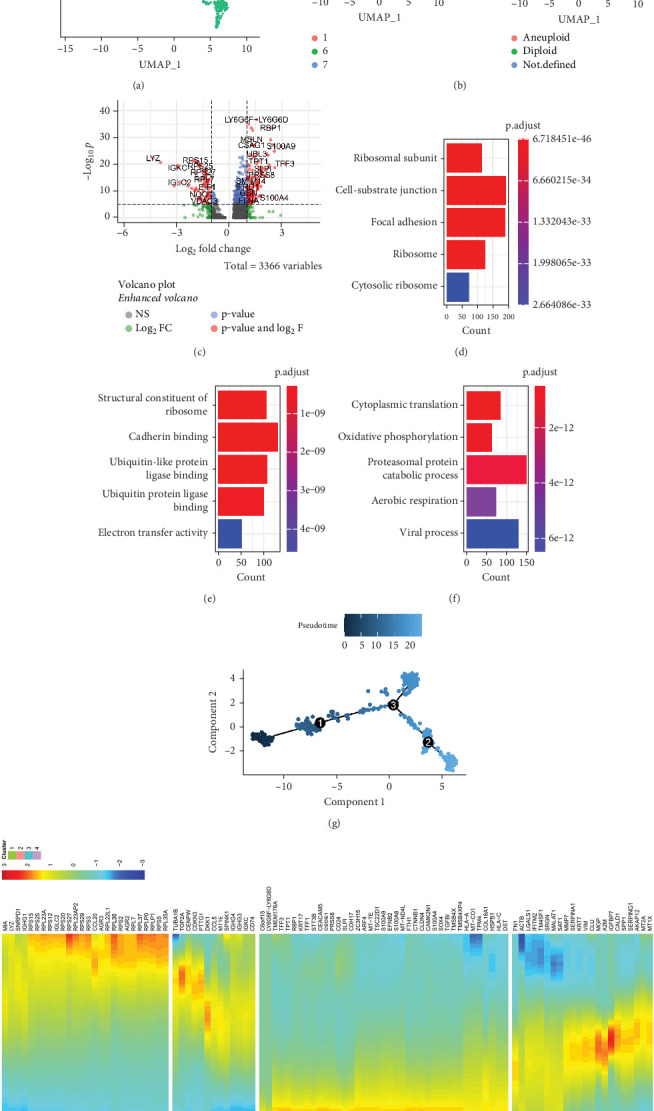
ScRNA-seq uncovered key genes for metastasis in CRC tumors. (a) UMAP visualization of cells from patients with metastasis in CRC. Cells were categorized into five cell types including B cells, CD8^+^ T cells, macrophages, monocytes, and epithelial cells. (b) UMAP visualization of epithelial cell subpopulations. These cells were categorized into three clusters (Clusters 1, 6, and 7) (left) or identified as aneuploid and diploid (right). (c) A volcano plot of DEGs between Cluster 7 and Clusters 1 and 6. (d, e, f) Enrichment analysis demonstrated the biological processes associated with DEGs in liver metastasis samples. (g) Trajectory analysis using Monocle 2. (h) Heatmap of top 10 genes for pseudotime trajectory analysis.

To explore tumor cell plasticity during the EMT process, we used scRNA-seq data from patients with metastasis of CRC in the GSE234804 dataset. According to the parameter settings, 1627 cells were identified (Figure S2(a) and S2(b)), and the top 2000 highly variable genes were identified (Figure S2(c)). Eight CRCs were chosen for further analysis (Figure S2(d) and S2(e)). Based on typical gene expression patterns, cells were classified into five cell types, including B cells, CD8^+^ T cells, macrophages, monocytes, and epithelial cells (Figure 4a). Since epithelial cell subpopulations were dominant in these single cells, we further categorized them into three cell subpopulations (Clusters 1, 6, and 7) (Figure 4b). Simultaneously, the epithelial cell subgroups (Clusters 1, 6, and 7) were identified as aneuploid and diploid cells by CopyKAT. The results revealed that concurrent Cluster 7 served as a subpopulation enriched with aneuploid cells, whereas Clusters 1 and 6 served as a subpopulation enriched with diploid cells (Figure 4b). We compared gene expression between the aneuploid cell subpopulations (Cluster 7) and diploid cell subpopulations (Clusters 1 and 6). In total, 3366 DEGs were identified (Figure 4c). Subsequent enrichment analysis of these DEGs revealed that their functions were correlated with “ribosomal subunit,” “structural constituent of the ribosome,” and “cytoplasmic translation” (Figures 4d, 4e, and 4f).

Meanwhile, we used Monocle 2 for the analysis, and the results showed distinct clusters across pseudotime (Figure 4g). We characterized the changes and trajectories of node genes during metastatic tumorigenesis by defining root cells in evolutionary Relationship 1. The heatmap showed the first 100 key genes that regulate metastatic CRC carcinogenesis (Figure 4h).

### 3.4. Determination of Key Genes Associated With the Development of Primary to Metastasis of CRC by ScRNA-Seq

**Figure 5:**
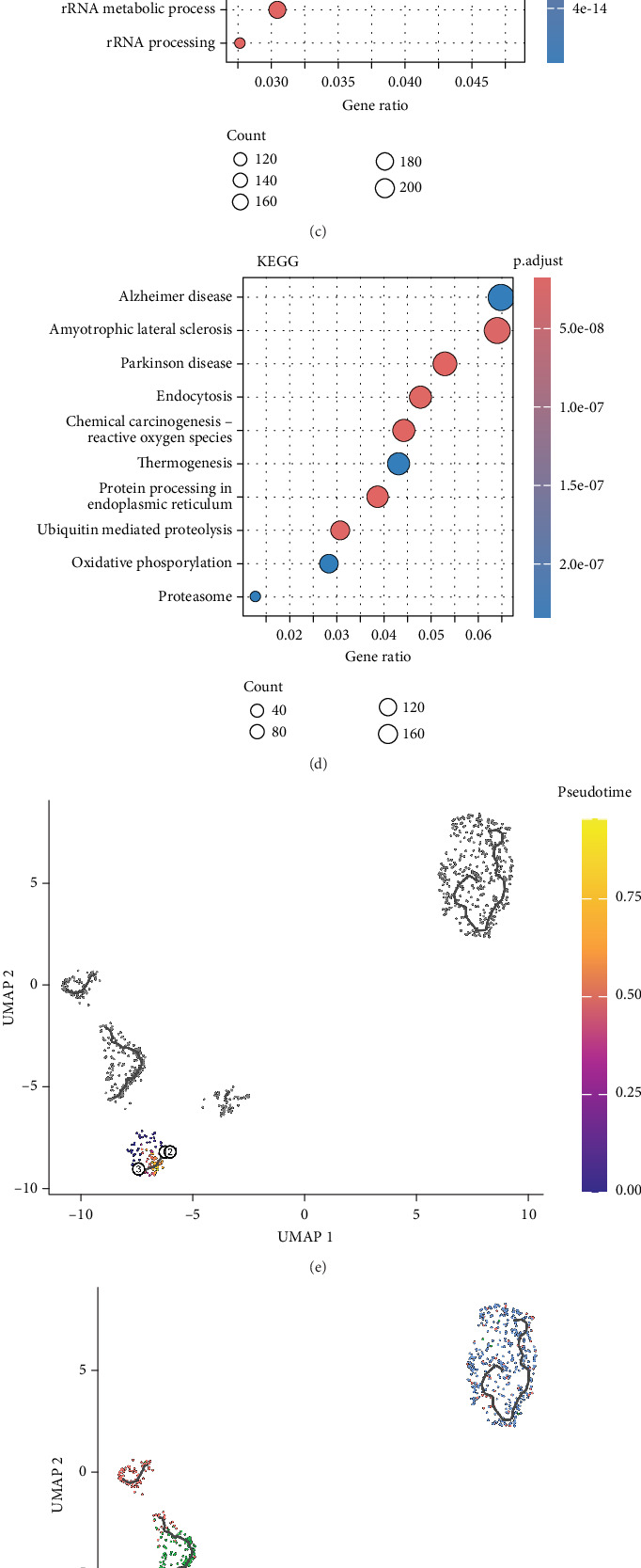
Determination of key genes associated with the development of primary cancer and metastatic carcinoma by scRNA-seq. (a) UMAP visualization of cells in primary CRC, EMT cells, and metastasis groups. (b) Heatmap of DEGs between primary and metastasis groups. (c) GO analysis demonstrated the biological processes associated with DEGs in liver metastasis samples. (d) KEGG analysis demonstrated the enrichment pathway of DEGs in liver metastasis samples. (e, f) Trajectory analysis of primary and metastasis subpopulations using Monocle 3. (g) The expression of top 10 genes in primary and metastasis subpopulations. (h) Heatmap of top 10 genes for pseudotime trajectory analysis.

To dynamically observe plasticity and heterogeneous changes during tumor aggravation, scRNA-seq data of primary and metastatic tumor cells were merged (Figure 5a). Furthermore, we compared gene expression in primary tumor cells and metastatic tumor cells and identified a total of 5470 DEGs. We identified the top 10 DEGs expressed in a heatmap (Figure 5b). These DEGs were subsequently analyzed using GO and KEGG enrichment analyses. We found that mRNA splicing and protein processing in the ER were altered during the development of primary tumors into metastatic tumors (Figure 5c,d). To further analyze the evolutionary spectrum of CRC metastasis, we defined the developmental trajectory of root cells in primary tumors and calculated the evolutionary relationship between primary carcinoma and metastasis using pseudotime trajectory analysis (Figure 5e,f). In the process of analyzing evolutionary relationships, we presented the nodes where gene expression changes occurred along with metastasis as UMAP plots. SMC3, EIF3L, BAG-1, TACC1, CNBP, heterogeneous nuclear ribonucleoprotein C1/C2 (HNRNPC), NIP7, HPRT1, CYP2S1, and FAM3B were identified as key metastatic node genes (Figure 5g,h).

### 3.5. Construction of a Prognostic Model

**Figure 6:**
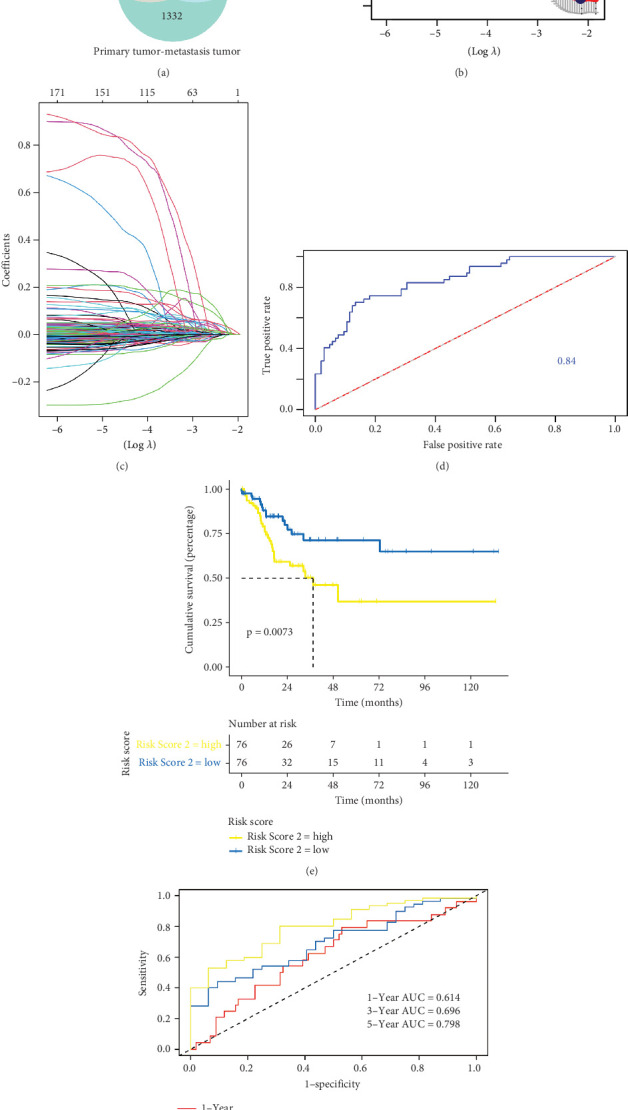
Construction of a prognostic model. (a) Venn diagnosis of DEGs for the three stages of tumor progression. (b) Tenfold cross-validation of adjusted parameter choices in lasso regression. (c) Log (*λ*) change curves of regression coefficients. (d) The ROC curves of five-gene model. (e) K-M curve results of the prognostic model of five genes. (f) ROC curve results prognostic model.

Based on the above results, we obtained plasticity gene modules for three stages of tumor progression: normal epithelial tumor, epithelial–mesenchymal tumor, and primary tumor–metastatic tumor. A total of 10,637 key genes were identified by taking the intersection of the three gene modules through a Venn analysis (Figure 6a). The clinical information of patients with the above 10,637 genes from TCGA-COAD dataset was integrated, extracted, and constructed a lasso regression model for establishment of tumor progression- and metastasis-related signatures (Figures 6b, 6c, and 6d). We have identified KRT20, FKBP9L, CCDC108, SIRT3, and ZFAND2A as significantly associated with patient prognosis. Kaplan–Meier (K-M) analysis demonstrated that individuals with high-risk scores had shorter overall survival (OS) than those with low-risk scores (Figure 6e). The AUC values of ROC curves for OS at 1, 3, and 5 years exceeded 0.61 (Figure 6f).

### 3.6. Predictive Gene Validation in CRC

**Figure 7:**
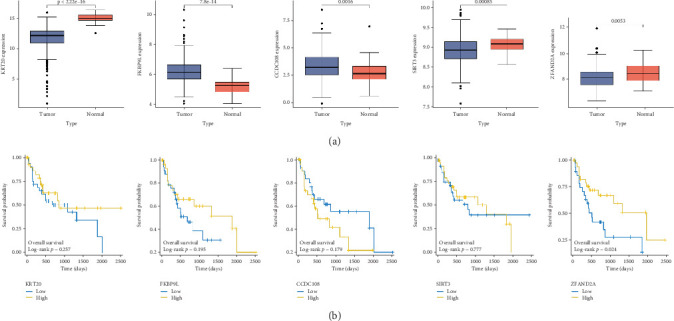
Predictive gene validation in CRC. (a) The expression levels of six genes between normal and tumor tissues in TCGA database. (b) K-M curves showing OS of patients with CRC grouped according to the expression levels of key genes.

We used the above five genes from TCGA-COAD data to predict gene expression and prognosis. FKBP9L and CCDC108 were found to be significantly increased in tumor tissues, whereas the expression of KRT20, ZFAND2A, and SIRT3 was significantly decreased in tumors (Figure 7a). To confirm the credibility of the logistic regression model in predicting tumor aggravation, we performed prognostic analysis of these five genes. The results revealed that high ZFAND2A expression prolonged the survival of patients with CRC patients (*p* = 0.024) (Figure 7b). To prove the independent predictive value of ZFAND2A, a nomogram was constructed. These findings suggested ZFAND2A as an independent prognostic factor (Figure S3). Therefore, we speculated that ZFAND2A might disrupt the tumor progression process during CRC development.

### 3.7. Overexpression of ZFAND2A Inhibited Proliferation, Migration, and Invasion of CRC Cells

**Figure 8:**
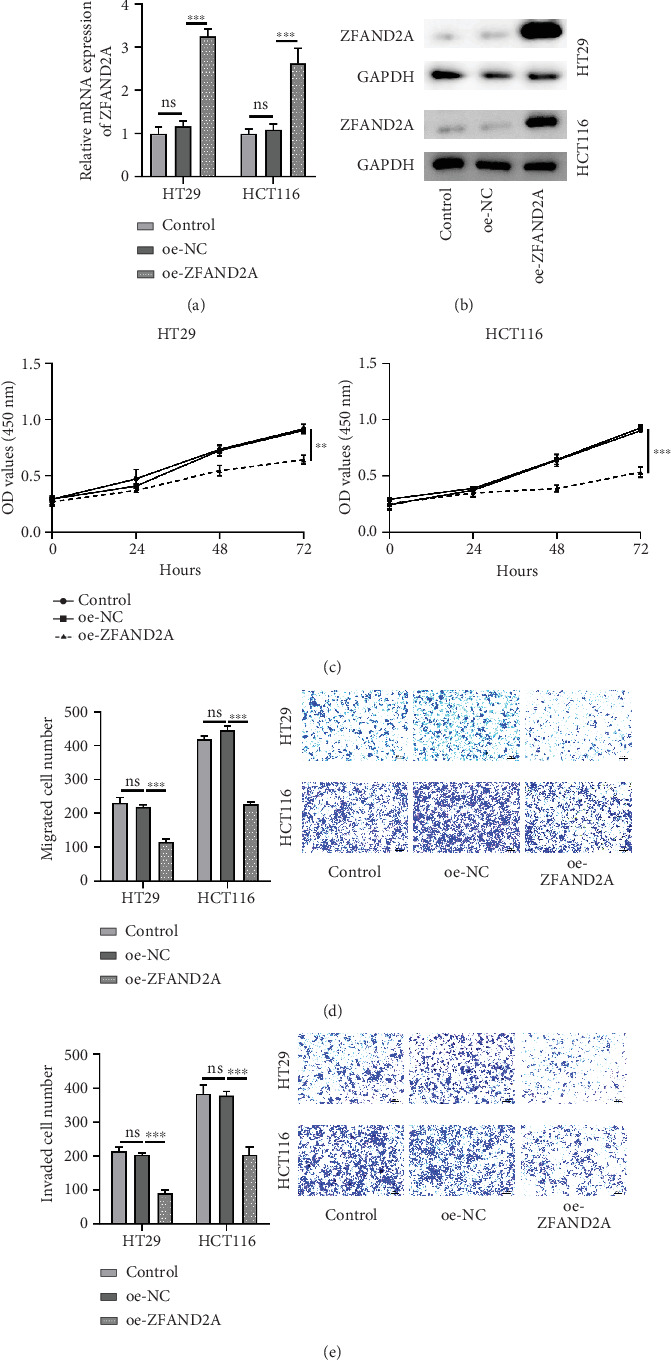
Overexpression of ZFAND2A inhibited proliferation, migration, and invasion of CRC cells. (a) The mRNA levels of ZFAND2A in HT29 and HCT116 cells were detected by qRT-PCR. (b) The protein levels of ZFAND2A in HT29 and HCT116 cells were detected by WB analysis. (c) Cell viability in HT29 and HCT116 cells was detected by CCK-8. The (d) migration and (e) invasion ability of HT29 and HCT116 cells was detected by transwell assay (all, *n* = 3). Data were shown as mean ± SD. ns represents no significant difference; ⁣^∗∗^*p* < 0.01 and ⁣^∗∗∗^*p* < 0.001.

To demonstrate this prediction, we constructed ZFAND2A overexpression cell lines by transfecting CRC cells (HT29 and HCT116) with a ZFAND2A plasmid vector or negative control vector. The results of qRT-PCR and WB indicated that the expression of ZFAND2A was increased in the ZFAND2A-transfected group compared with that in the control and NC groups (Figure 8a,b). This result revealed that the cell lines stably expressing ZFAND2A were successfully constructed. Furthermore, cell viability was assessed by CCK-8. The results revealed that an increased ZFAND2A expression significantly inhibited CRC cell viability (Figure 8c). Furthermore, transwell assay results showed that overexpression of ZFAND2A reduced the migration and invasion abilities of CRC cells (Figure 8d,e). In summary, these results suggest that ZFAND2A negatively regulates proliferation and migration of CRC cells and has an anticancer effect.

### 3.8. ZFAND2A Was Associated With Macrophage Infiltration and Promoted Macrophage M1 Polarization

In this study, polyploidy identification of all cell types from patients with primary CRC was performed using InferCNV in the preliminary stage. Interestingly, in addition to the tumor cells observed in this study, macrophages showed a large amount of variation on Chromosome 7 (Figure S4(a)). Therefore, we hypothesized that there are genes involved in the regulation of macrophage immune infiltration during CRC progression. ZFAND2A was also analyzed for immune infiltration. ZFAND2A expression showed a positive correlation with the immune infiltration of macrophages in CRC (Figure S4(b), S4(c), and S4(d)). These results suggest that ZFAND2A is associated with macrophage infiltration.

**Figure 9:**
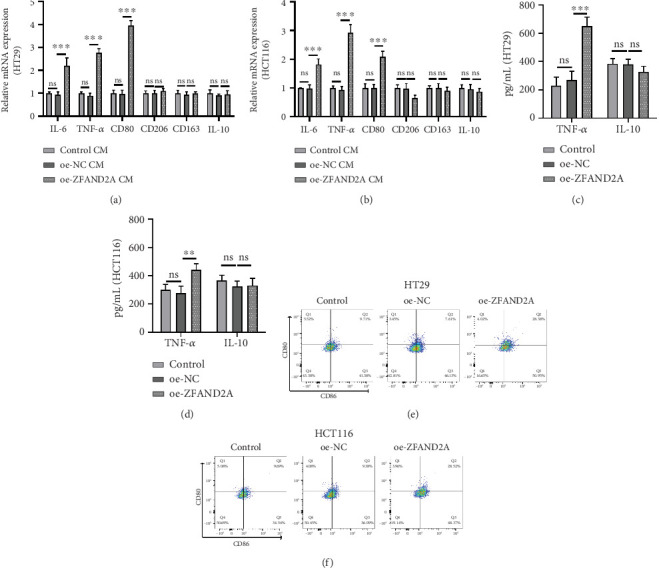
ZFAND2A promoted macrophage M1 polarization. (a, b) The mRNA levels of M1 (IL-6, TNF-*α*, and CD80) and M2 (CD206, CD163, and IL-10) markers in macrophages treated with (a) HT29- and (b) HCT116-cell media of different treatment groups were detected by qRT-PCR. (c, d) The expression levels of TNF-*α* and IL-10 in macrophages treated with (c) HT29- and (d) HCT116-cell media of different treatment groups were detected by ELISA. (e, f) Flow cytometry determining the percentage of CD80^+^ and CD86^+^ cells (all, *n* = 3). Data were shown as mean ± SD. ⁣^∗∗^*p* < 0.01 and ⁣^∗∗∗^*p* < 0.001.

Subsequently, we validated the predictive results of immune infiltration. Macrophages were cocultured with different cell media (CM) from CRC cells (control, NC, or ZFAND2A). Macrophages cocultured with CM from overexpressed ZFAND2A CRC cells significantly increased M1 markers (IL-6, TNF-*α*, and CD80) expression. In contrast, ZFAND2A overexpression barely affected the expression of M2 markers (CD206, CD163, and IL-10) (Figure 9a,b). In addition, the levels of macrophage polarization markers were analyzed using ELISA. Overexpression of ZFAND2A enhanced TNF-*α* expression. However, it did not alter the expression of IL-10 (Figure 9c,d). Flow cytometry results suggested that overexpression of ZFAND2A showed significant higher expression of M1 macrophage-related cell surface marker, namely, CD86 and CD80 (Figure 9e,f). Taken together, the above results demonstrate that ZFAND2A promotes macrophage M1 polarization.

## 4. Discussion

Distal metastasis of CRC is a multistep process that is fatal in most case [34]. In this study, we propose a dynamic model of tumor heterogeneity involving epithelial-to-tumor cell evolution, mesenchymal-to-tumor cell evolution, and CRC liver metastatic processes.

CRC originates in the intestinal epithelium of the colon and rectum and is influenced by a variety of factors, including genetics, the environment, and chronic inflammation [35]. There is a consensus that frequent genetic alterations in initiating epithelial cells are strongly associated with CRC progression. Some studies have found that normal epithelial adenomatous polyps with chromosomal instability or microsatellite instability eventually progress to CRC [36, 37]. To determine key node genes for the evolution of initiating epithelial cells into tumors, we analyzed scRNA-seq data from the GSE234804 database and identified epithelial cell subpopulations. CopyKAT was used to identify aneuploid and diploid cells in epithelial cell subpopulations. Choi et al. found that high-grade atypical hyperplastic adenomas exhibited aneuploidy and could develop into CRC [38]. Therefore, we believe that epithelial cells in the aneuploid subpopulation have the potential to develop into CRC. In this study, pseudoannotation was performed to obtain epithelial tumor evolutionary node gene modules during colon carcinogenesis.

During the evolution of CRC metastasis, the external environment induces EMT and epithelial plasticity [39]. Upon the EMT activation, tight junctions in tumor cells are dissolved, apical–basal polarity is disrupted, and cytoskeletal structures are reorganized. These physical changes promote cell migration away from the original site, invasion into adjacent tissues, metastasis, survival in the bloodstream, and, ultimately, metastatic tumor growth in distant organs [40, 41]. Therefore, we need to understand the key node genes that took part in the evolution of the epithelial–mesenchymal phenotype. We identified 10 genes associated with tumor epithelial–mesenchymal evolution (EMT). Among them, SMC3, as a member of the SMC protein family [42], has been found to have something to do with the formation of colorectal brain metastasis [43]. BAG-1, as an antiapoptotic protein, is highly expressed in CRC tissue [44]. BAG-1 is crucial for promoting the survival of CRC cells by acting as a selective regulator of p50-p50 NF-*κ*B responsive genes in CRC cells [45]. HNRNPC belongs to RNA-binding protein family. SNHG3 may enhance the translocation of HNRNPC into the nucleus to promote CRC progression [46]. RALYL potentially exerts an inhibitory effect on CRC by engaging with HNRNPC to orchestrate the alternative splicing of MNK2 [47]. These genes can serve as nodal genes to determine the mesenchymal status of the epithelial cells. In addition, the key genes involved in CRC metastasis have been explored. The scRNA-seq data of patients with liver metastasis from the GSE234804 database were used for analysis. The results revealed 100 key genes that regulate metastatic CRC carcinogenesis.

To this point, we have obtained the genes that characterize the three stages of CRC aggradation: normal epithelial-like tumor cell evolution, epithelial–mesenchymal phenotypic cell-to-tumor conversion, and primary tumor metastasis evolution. In this regard, we integrated the key genes in these three stages and obtained 10,637 genes for the evolution of CRC aggravation and then constructed the lasso regression model. We found that poorly expression of ZFAND2A in CRC tissues was associated with poor prognosis of CRC. We also verified that ZFAND2A inhibited CRC cell proliferation and migration. However, the role of ZFAND2A in tumors is not well understood. ZFAND2A knockdown regulates the proliferation of melanoma cells [48]. The results of this study further prove that ZFAND2A plays a significant inhibitory role in the proliferation, migration, and invasion of cancer cells.

In the present study, ZFAND2A was also shown to correlate with macrophage infiltration. In contrast, overexpression of ZFAND2A promoted the M1 polarization of macrophages without altering the M2 polarization process. It is well known that macrophages are divided into classically activated macrophages (M1, which are used to kill tumor cells) and selectively activated macrophages (M2, which are used to promote tumor cells) [49]. TAMs are macrophages that are abundantly present in the TME, and TAM mainly is M2 macrophages [50, 51]. We found that ZFAND2A increased M1 macrophages, suggesting that ZFAND2A plays a role in killing tumor cells by promoting the M1 phenotype. Although the effect of ZFAND2A on tumors is still lacking research, previous studies [48] have found that bortezomib significantly induces the expression of ZFAND2A in human melanoma. The downregulation of ZFAND2A could lead to sensitization to bortezomib, while the upregulation of ZFAND2A could protect melanoma cells from the drug invasion. The downregulation of ZFAND2A hinders the clonal potential and spheroid growth of melanoma, promoting caspase activation and apoptotic cell death in the absence of drugs. The research results confirm that ZFAND2A is a potential therapeutic target for melanoma, while also highlighting the translational potential of ZFAND2A as a cancer therapeutic target.

Although the results of this study are noteworthy, there are still some shortcomings. First, the molecular mechanism of ZFAND2A in CRC immune infiltration and metastasis and the mechanistic pathway of how ZFAND2A overexpression in tumor cells affect macrophages need to be verified experimentally. And, in the flow cytometry experiment to verify macrophage polarization, we did not detect the markers of M2 macrophages. In addition, there is a lack of exploration of other immune cell subtypes and their interaction with ZFAND2A. Second, validation of ZFAND2A in vivo experiments could help to verify its role in CRC progression and metastasis. Third, the TME of CRC consists of complex components, and this study was based on a three-stage evolutionary model of tumor deterioration. Therefore, this model has certain limitations.

## 5. Conclusion

In conclusion, key genes at the three stages of CRC heterogeneity change were explored by analyzing two scRNA-seq datasets. These genes include key nodal genes during epithelial tumor cell evolution, differentiation of epithelial–mesenchymal subpopulations to tumor cells, and tumor metastasis. In addition, we integrated these nodal genes to identify gene modules associated with the progression and metastasis of CRC. Among them, ZFAND2A was screened as a key signature gene during colorectal carcinogenesis. Further analyses revealed a correlation between ZFAND2A expression and macrophage infiltration. ZFAND2A is a therapeutic target for CRC metastasis.

## Data Availability

The authors confirm that the data supporting the findings of this study are available within this article and its supporting information or available upon request without undue reservation.

## Supporting Information

Supporting Information Additional supporting information can be found online in the Supporting Information section. Table S1: List of all primers used in qRT-PCR. Figure S1: scRNA-seq data revealed 20 cell clusters with diverse annotations and heterogeneous expression patterns in GSE261012 and GSE234804. (a, b) A total of 7944 cells were identified. (c) Diagram showing variations in gene expression levels in all CRC cells. The red dots represent genes with highly variable expression, and the black dots represent genes with stable expression. (d) PCA clearly separated individual CRC cells. (e) Dimension reduction analysis. (f) Heatmaps of relative copy number ratios of primary CRC tumor cells inferred by CopyKAT. Figure S2: ScRNA-seq data revealed eight cell clusters with diverse annotations and heterogeneous expression patterns in GSE234804. (a, b) A total of 1627 cells were identified. (c) Diagram showing variations in gene expression levels in all CRC cells. The red dots represent genes with highly variable expression, and the black dots represent genes with stable expression. (d) PCA clearly separated individual CRC cells. (e) Dimension reduction analysis. Figure S3: Construction of a nomogram and analysis of key biological characteristics. Figure S4: Association between ZFAND2A and immune cell infiltration. (a) Aneuploidy characterization of all cell types in patients with primary CRC cancer by InferCNV. (b) Histogram showing the percentages of immune cells in each sample. (c) Proportions of different types of infiltrating immune cells in the high- and low-risk groups. (d) Risk scores for different types of immune cells.
