# Supplementary material for: Single-Cell RNA-Seq Recognized Key Genes for Metastasis and Macrophage Infiltration in Colorectal Cancer
Source: Hum Mutat. 2025 May 15;2025:9488531. doi: 10.1155/humu/9488531 (PMC12097859; doi:10.1155/humu/9488531)
Supplement: Supporting Information — Additional supporting information can be found online in the Supporting Information section. Table S1: List of all primers used in qRT-PCR. Figure S1: scRNA-seq data revealed 20 cell clusters with diverse annotations and heterogeneous expression patterns in GSE261012 and GSE234804. (a, b) A total of 7944 cells were identified. (c) Diagram showing variations in gene expression levels in all CRC cells. The red dots represent genes with highly variable expression, and the black dots represent genes with stable expression. (d) PCA clearly separated individual CRC cells. (e) Dimension reduction analysis. (f) Heatmaps of relative copy number ratios of primary CRC tumor cells inferred by CopyKAT. Figure S2: ScRNA-seq data revealed eight cell clusters with diverse annotations and heterogeneous expression patterns in GSE234804. (a, b) A total of 1627 cells were identified. (c) Diagram showing variations in gene expression levels in all CRC cells. The red dots represent genes with highly variable expression, and the black dots represent genes with stable expression. (d) PCA clearly separated individual CRC cells. (e) Dimension reduction analysis. Figure S3: Construction of a nomogram and analysis of key biological characteristics. Figure S4: Association between ZFAND2A and immune cell infiltration. (a) Aneuploidy characterization of all cell types in patients with primary CRC cancer by InferCNV. (b) Histogram showing the percentages of immune cells in each sample. (c) Proportions of different types of infiltrating immune cells in the high- and low-risk groups. (d) Risk scores for different types of immune cells. [file 9488531.f1.zip › Table S1.docx]

Table S1: List of all primers used in qRT-PCR

| Primer | Forward sequence (5′-3′) | Reverse sequence (5′-3′) |
| --- | --- | --- |
| ZFAND2A | GATCATTTTCCATACGCTGCAC | CGTCTGGTATCTGGCCCTTTT |
| IL-6 | TCGGTCCAGTTGCCTTCTC | GAGGTGAGTGGCTGTCTGTG |
| TNF-α | CCCTCACACTCAGATCATCTTCT | GCTACGACGTGGGCTACAG |
| CD80 | TCTCAGAAGTGGAGTCTTACCCT | GATTGGAGGGTGTTCCTGGG |
| CD206 | CCAAACGCCTTCATTTGCCA | ACCTTCCTTGCACCCTGATG |
| CD163 | CCGGGAGATGAATTCTTGCCT | GGTATCTTAAAGGCTCACTGGGT |
| IL-10 | AGGGCACCCAGTCTGAGAAC | TCTTCACTCTGCTGAAGGCAT |
| GAPDH | AAAGCCTGCCGGTGACTAAC | AGGAAAAGCATCACCCGGGAG |
